# Myrtaceae Plant Essential Oils and their β-Triketone Components as Insecticides against *Drosophila suzukii*

**DOI:** 10.3390/molecules22071050

**Published:** 2017-06-24

**Authors:** Chung Gyoo Park, Miyeon Jang, Eunsik Shin, Junheon Kim

**Affiliations:** 1 Institute of Agriculture and Life Science, Gyeongsang National University, Jinju 52828, Korea; parkcg@gnu.ac.kr; 2 Division of Applied Life Science (BK21+ Program), Gyeongsang National University, Jinju 52828, Korea; tama4751@naver.com (M.J.); tls890624@gmail.com (E.S.); 3 Forest Insect Pests and Diseases Division, National Institute of Forest Science, Seoul 02455, Korea

**Keywords:** spotted wing drosophila, manuka, kanuka, triketones

## Abstract

Spotted wing drosophila (SWD, *Drosophila suzukii* (Matsumura), Diptera: Drosophilidae) is recognized as an economically important pest in North America and Europe as well as in Asia. Assessments were made for fumigant and contact toxicities of six Myrtaceae plant essential oils (EOs) and their components to find new alternative types of insecticides active against SWD. Among the EOs tested, *Leptospermum citratum* EO, consisting mainly of geranial and neral, exhibited effective fumigant activity. Median lethal dose (LD_50_; mg/L) values of *L. citratum* were 2.39 and 3.24 for males and females, respectively. All tested EOs except *Kunzea ambigua* EO exhibited effective contact toxicity. LD_50_ (µg/fly) values for contact toxicity of manuka and kanuka were 0.60 and 0.71, respectively, for males and 1.10 and 1.23, respectively, for females. The LD_50_ values of the other 3 EOs-*L*. *citratum*, allspice and clove bud were 2.11–3.31 and 3.53–5.22 for males and females, respectively. The non-polar fraction of manuka and kanuka did not show significant contact toxicity, whereas the polar and triketone fractions, composed of flavesone, isoleptospermone and leptospermone, exhibited efficient activity with the LD_50_ values of 0.13–0.37 and 0.22–0.57 µg/fly for males and females, respectively. Our results indicate that Myrtaceae plant EOs and their triketone components can be used as alternatives to conventional insecticides.

## 1. Introduction

The spotted wing drosophila (SWD, *Drosophila suzukii* (Matsumura), Diptera: Drosophilidae), is indigenous to South-eastern Asia. It has invaded and spread across North America and Europe and most recently, has been found in South America [1,2,3]. Unlike other closely related *Drosophila* species, SWD can lay eggs with a serrated ovipositor on maturing and undamaged healthy thin-skinned fruits and inflict substantial economic losses, especially to blueberry, cherry and raspberry [4,5,6]. Developing maggots accelerate fruit softening and decomposition, rendering fruits unmarketable. Current control methods for SWD mainly depend on application of conventional insecticides such as pyrethroids, organophosphates, spinosyns, and neonicotinoids [7,8]. Unfortunately, frequent application of the conventional insecticides is creating public concerns due to their adverse effects on the environment and human health. As a result, there is growing interest in finding less ecologically damaging SWD control methods, such as natural enemies [9] and biopesticides [10,11,12,13], and a strong push to develop new, organic and ecologically sustainable control methods for this destructive pest. Plant essential oils (EOs) could be an eco-friendly alternative to chemical insecticides as they have been reported to have an array of bioactivities, including insecticidal, repellent, and feeding and oviposition deterrent activities for control of a range of insect species [14,15,16]. Other advantages of volatile plant EOs as eco-friendly biopesticides include commercial availability, low cost, multiple modes of action, low toxicity to vertebrates, and brief persistence in the soil [17,18,19,20,21]. The insecticidal activity of EOs against SWD has been investigated [10,11,12,13]. In this study, we assessed the insecticidal activity of Myrtaceae plant EOs and their component β-triketones against adult SWD to find new types of alternatives to current insecticides. Myrtaceae plant EOs were selected because they are known to have insecticidal and repellent activities [10,22,23] and, thus, were assumed to have effective insecticidal activity against SWD.

## 2. Results

### 2.1. Chemical Analyses of Active EOs

The chemical composition of a fumigant-active EO, *Leptospermum citratum*, and two contact toxicity active EOs, *L. ericoides* (kanuka), and *L. scoparium* (manuka), are shown in Table 1. Similar to the previous reports [24,25], geranial (33.4%), citronellal (22.8%) and neral (17.8%) were identified as the major components of *L. citratum* EO. 

In contrast, kanuka and manuka EOs consisted of mainly sesquiterpenes (38.5% and 52.0%, respectively) and triketones (27.6% and 34.4%, respectively) and the results were in line with the previous report [27]. The triketones in both kanuka and manuka EOs consisted of flavesone (**1**), isoleptospermone (**2**) and leptospermone (**3**).

### 2.2. Fumigant Activity of EOs and their Major Components

Among the six tested EOs, only one EO from *L. citratum* showed 98.0% and 94.0% mortality at a concentration of 11.76 mg/L air against males and females, respectively. In contrast, others showed 0–30.0% and 4.0–16.0% mortality at the same concentration. Median lethal concentration (LC_50_) values of *L. citratum* EO were estimated at 2.39 and 3.24 mg/L air against males and females, respectively (Table 2). The LC_50_ values of the major components geranial, citronellal and neral have been previously reported [10]; therefore, we did not test the fumigant activities of each component individually. 

### 2.3. Contact Toxicity of EOs and Their Major Components

At a concentration of 20 µg/fly, all the tested EOs showed 93–100% male mortality and 98–100% female mortality, with the exception of *Kunzea ambigua* (61.2%). Kanuka and manuka EOs exhibited 97.9–100% contact toxicity against males and 100% against females at a concentration of 2.5 µg/fly, whereas other EOs showed contact toxicity rates of 14.9–55.3% and 9.9–19.6% against males and females, respectively, at the same concentration. Among the tested EOs, the median lethal dose (LD_50_) values of kanuka and manuka EOs against males and females were the lowest. The LD_50_ value of kanuka EO was estimated at 0.71 and 1.23 µg/fly against males and females, respectively, and the LD_50_ of manuka EO was 0.60 and 1.10 µg/fly, respectively (Table 3). Clover oil and allspice EOs had the next highest levels of toxicity. *K. ambigua* EO showed the lowest contact toxicity in terms of LD_50_ value. 

Silica gel chromatography of kanuka and manuka EOs gave good separation into a non-polar fraction consisting mainly of sesquiterpene hydrocarbons and a polar fraction that consisted largely of triketones. Further fractionation of polar fraction showed that triketones composed 97.1% of the triketone fraction.

The non-polar fraction of kanuka and manuka EOs did not show significant insecticidal activity, whereas the polar and triketone fractions exhibited significantly higher activity than whole oils (Table 3 and Table 4). The triketone fraction also exhibited higher activity than that of polar fraction in terms of LD_50_ value (Table 4).

## 3. Discussion

*Leptospermum citratum* showed fumigant activity and contact toxicity against adult SWD. Eucalyptus oils have been reported to have insecticidal activity [28]. Among the eucalyptus oils, *Melaleuca teretifolia* EO, which is composed mainly of geranial and neral, exhibited fumigant and contact toxicity against adult SWD [10]. LC_50_ and LD_50_ values of *L. citratum* for fumigant and contact toxicity, respectively, were similar to those of *M. teretifolia*. The composition of *L. citratum* was also similar to *M. teretifolia*. Therefore, it can be concluded that the toxicity of these EOs may come from geranial and neral. 

In contact toxicity tests, the EOs were relatively effective, except for *Kunzea ambigua* EO. In terms of LD_50_ values, allspice and clove bud showed similar activity to the previously reported active EOs [10,11,12]. The EOs from allspice and clove bud were reported to consist of thymol [29,30] and eugenol [31], respectively. Thymol is known to have contact toxicity against SWD with an LD_50_ value of 1.73 µg/fly [11]. Although the contact toxicity of eugenol against SWD was not tested in this study, contact toxicity of eugenol against insect pests is well known [31,32,33]. Activity of thymol and eugenol may be attributed to the contact toxicity of allspice and clove bud, respectively.

Kanuka and manuka EOs were the most active EOs in contact toxicity against SWD compared to previously reported ones [10,11,12,34]. The lack of activity in the non-polar fraction of kanuka and manuka EOs, which contained the hydrocarbons monoterpene and sesquiterpene, clearly showed that the activity is associated with the polar components of oils. The activity of the triketone fraction indicated that the toxicity is related to the presence of triketones, flavesone (**1**), isoleptospermone (**2**) and leptospermone (**3**). Kanuka and manuka EOs and their triketone components are reported to have antimicrobial [27,35], antiviral [36], and acaricidal activities [37,38]. To the best of our knowledge, this is the first report describing the insecticidal activity of β-triketones isolated from kanuka and manuka EOs. Leptospermone, a β-triketone, is a natural product used as an herbicide [39] and inhibits *p*-hydroxyphenylpyruvate dioxygenase, an enzyme involved in plastoquinone synthesis, as a molecular target site [40]. It is not clear whether the contact toxicity caused by β-triketones is associated with the same mode of action as occurs in the plant; this was not addressed extensively in our study. The β-triketones responsible for contact toxicity against SWD possess multiple carbonyl groups on a six-membered ring (cyclohexane), and this structure is rare in natural phytotoxins. Many derivatives of leptospermone, such as nitisinone and sulcotrione, have been synthesized and selected as herbicides [41]. Some new derivatives of β-triketones with new modes of action, as envisaged by this experiment, are also expected to be used as novel insecticides. 

Fumigant activity of dichlorvos and contact toxicity of cypermethrin assessed during these experiments were similar to those previously reported [10,11]. Even though both dichlorvos and cypermethrin are more effective against SWD than the EOs and their components, they have high mammalian toxicity and therefore were expected to have much higher non-target hazards than the EOs and their components.

## 4. Materials and Methods 

### 4.1. Insects

The colony of SWD was initially obtained from Chonnam National University (Gwangju, Korea) and has been successively maintained in the Insect Chemical Ecology Laboratory, Gyeongsang National University. The colony was maintained in a netted cage (25 × 25 × 20 cm^3^, BugDorm, Taiwan) with an artificial diet for larvae and 50% sugar solution for adults at 24–26 °C, 60–70% RH and a photoperiod of 16:8 (L:D) [42]. Five- to 7-day-old adults were used for bioassays.

### 4.2. Chemicals and Fractionation of Essential Oils

Essential oils (EOs) used in this bioassay are listed in Table 5. Six Myrtaceae plant EOs were obtained from Oshadhi Ltd. (Cambridge, England) and La Drome (Die, France). Wakogel C-200 (Wako Pure Chemical, Osaka, Japan) was used for chromatography. Dichlorvos (DDVP), and cypermethrin were purchased from Sigma-Aldrich (St. Louis, MO, USA).

**Figure 1:**
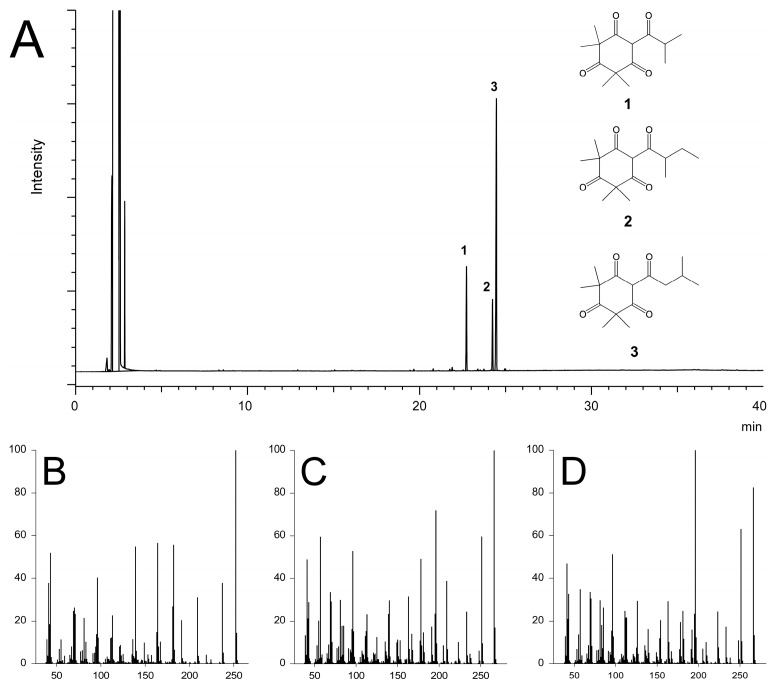
Gas chromatogram of the triketone fraction and structures of triketones (**A**) and mass spectra (**B–D**). **1**: flavesone (**B**); **2**: isoleptospermone (**C**); **3**: leptospermone (**D**).

Non-polar and polar fractions of kanuka and manuka EOs were prepared as follows: a sample of oil (5 g) was loaded onto a column of activated silica gel (Wakogel C-200) and eluted with hexane and then with diethyl ether to yield non-polar and polar fractions. The triketone fraction was prepared by further fractionation of the polar fraction with 5% diethyl ether in hexane (Figure 1). The solvent was removed using a rotary evaporator and the fractions were dried and stored at 4 °C before analysis and testing. 

### 4.3. Instrumental Analysis

Gas chromatography (GC) analysis was performed using a GC-17A (Shimadzu, Kyoto, Japan) equipped with a flame ionization detector (FID). A DB-5MS column (30 m × 0.25 mm i.d., 0.25 μm film thickness; J&W Scientific, Folsom, CA, USA) was used for separation of the analytes. GC-mass spectrometry (GC-MS) analysis was performed on a GC-2010 coupled with GCMS-QP2010 plus (Shimadzu) using an HP-Innowax column (30 m × 0.25 mm i.d., 0.25 μm film thickness; J&W Scientific). The oven temperature for GC and GC-MS analyses was programmed as follows: isothermal at 40 °C for 1 min, rose to 250 °C at a rate of 6 °C/min, and was held for 4 min. The injector temperature of GC-FID and GC-MS was 250 °C. The detector temperature of the GC-FID was set at 280 °C. The temperatures of the transfer line and ion source for GC-MS were 250 °C and 230 °C, respectively. One microliter of 5000 ppm EOs dissolved in hexane was injected with a split ratio of 1:50. Each EO was analyzed three times. Helium was used as carrier gas at a flow rate of 1.5 mL/min for GC and of 1.0 mL/min for GC-MS. Most of the components of the EOs were identified by comparing the mass spectra of each peak with those of authentic samples in the NIST/EPA/NIH MS library (Gaithersburg, MD, USA) and by comparison of retention indices determined on two different columns with those of authentic compounds. Flavesone (**1**), isoleptospermone (**2**) and leptospermone (**3**) were identified by comparison of retention indices and mass spectra with previous reports [43,44,45].

### 4.4. Fumigant Toxicity Assay

For fumigant toxicity assays, a glass cylinder (11 cm in height, 4.5 cm inner diameter; 170 mL, with a sieve placed in the middle) was used. EOs and DDVP dissolved in acetone (20 µL) were applied to a paper disc. After a 10 min incubation to allow the acetone to evaporate, the paper disc was placed on the bottom lid of the cylinder. The concentration range was 0.74–11.76 mg/L. Dichlorvos, an organophosphorus insecticide, was applied as a positive control in range of 0.07–73.5 µg/L. Acetone alone was used as a negative control. Twenty adult SWDs (10 males and 10 females) were placed on the sieve with a cotton wick soaked with 10% sugar solution, thereby preventing their direct contact with the test plant oils and compounds. The top and bottom lids were sealed with Parafilm to prevent fumigant leakage. The insects were maintained at 24–26 °C and 70% relative humidity. After 24 h treatment, they were moved to a new plastic Petri dish (4 cm in height, 9.6 cm diameter) and covered with a lid with a mesh–hole (4 cm diameter) for 10 min. The adult flies were considered dead if their appendages did not move after being touched with a fine brush. All treatments were replicated 5 times.

### 4.5. Contact Toxicity Assay

To test contact toxicity of EOs and their components, EOs (0.313–20 µg) and three fractions of EOs (0.078–10 µg) dissolved in acetone (1 µL) were topically applied to ventral abdomen using a micro syringe with a repeating dispenser (Hamilton, Reno, NV, USA). As a positive control, cypermethrin, a pyrethroid insecticide, was applied as above at a range of 0.025–50 ng/fly. After application, the adults were placed in a plastic Petri dish (4 cm in height, 9.6 cm diameter) with a cotton wick soaked in 10% sugar solution and covered with a lid which had a mesh-hole (4 cm diameter), thereby preventing fumigant effects of the tested EOs or fractions of EOs. After 24 h treatment, mortality was checked as above. Each treatment was performed 5 times with 20 adult SWDs (10 males and 10 females).

### 4.6. Statistical Analyses 

The corrected mortality was calculated using Abbott’s formula [46]. Probit analysis was used to estimate the LC_50_ values with dose-response data. Statistical analyses were performed using JMP ver. 9.0.2 (SAS Institute Inc., Cary, NC, USA). 

## 5. Conclusions

Kanuka and manuka EOs and their β-triketone components exhibited excellent contact toxicity against SWD. These are expected to be applied for protection of postharvest fruits. Considering that most insecticides currently in use are synthetic ones, the EOs from Myrtaceae and their components are quite promising and showing potential for the development of natural insecticides. However, further studies addressing the safety of these botanical insecticides to humans and host plants, their formulations, and their modes of action are necessary for practical use of plant EOs and their components as eco-friendly and novel SWD control agents.

## Figures and Tables

**Table 1:** GC-MS identification, RI values and % peak area contribution of active oil components.

Compound	RI Values ^1^	*L. citratum*	*L. ericoides*	*L. scoparium*
α-Pinene	934	-	19.9	1.4
β-Pinene	979	0.1	-	-
Myrcene	989	0.3	-	0.4
Limonene	1025	-	1.0	-
*p*-Cymene	1027	0.1	0.4	-
1,8-Cineole	1034	-	1.3	-
γ-Terpinene	1059	-	0.6	-
Linalool	1102	2.4	-	-
Citronellal	1155	22.8	-	-
Isopulegol	1162	3.2	-	-
Nerol	1228	0.4	-	-
Citronellol	1230	10.7	-	-
Neral	1242	17.8	-	-
Geraniol	1253	2.3	-	-
Geranial	1272	33.4	-	-
Citronellyl acetate	1350	1.1	-	-
α-Cubebene	1350	-	2.1	4.7
α-Copaene	1380	-	5.0	5.5
α-Gurjunene	1412	-	0.7	1.1
β-Caryophyllene	1426	-	1.4	3.0
6,9-Guaiadiene	1444	-	1.8	1.8
*trans*-Muurola-3,5-diene	1454	-	2.0	7.2
γ-Muurolene	1476	-	2.7	5.7
α-Selinene	1496	-	4.6	4.5
γ-Cadinene	1523	-	3.7	4.9
Calamenene	1528	-	13.9	13.6
Flavesone	1537	-	8.7	11.7
α-Copaene-11-ol	1539	-	0.6	-
Isoleptospermone	1615	-	4.9	5.5
Leptospermone	1627	-	14.0	17.2
Sum		94.6	89.3	88.1

^1^ RI (retention index) values were calculated following van Den Doold and Kratz on a non-polar column (DB-5MS) [26].

**Table 2:** LC_50_ values of fumigant essential oils active against SWD.

Essential Oil	LC_50_ (mg/L)	95% CL (mg/L)	Slope ± SE	Effect Test	
				χ^2^	*P*
**Male**					
*Leptospermum citratum*	2.39	1.42–3.440	4.34 ± 1.26	26.84	<0.0001
DDVP	0.24 × 10^−3^	0.04×10^−3^–0.50 × 10^−3^	1.44 ± 0.60	20.81	<0.0001
**Female**					
*Leptospermum citratum*	3.24	1.99–4.50	4.62 ± 1.37	28.34	<0.0001
DDVP	0.36 × 10^−3^	0.20 × 10^−3^–0.66 × 10^−3^	1.55 ± 0.90	22.38	<0.0001

CL: confidence limit.

**Table 3:** LD_50_ values of contact toxicity of essential oils against SWD.

Essential Oil	LD_50_ (µg/fly)	95% CL (µg/fly)	Slope ± SE	Effect Test	
				χ^2^	*P*
**Male**					
*Leptospermum citratum*	3.31	1.92–4.93	1.77 ± 0.50	25.19	<0.0001
*Leptospermum ericoides*	0.71	0.35–1.24	1.52 ± 0.53	26.96	<0.0001
*Leptospermum scoparium*	0.60	0.28–1.07	1.57 ± 0.59	24.37	<0.0001
*Kunzea ambigua*	7.34	na–11.92	1.28 ± 0.87	2.31	0.1287
*Pimenta dioica*	2.26	1.25–3.61	2.14 ± 0.78	22.24	<0.0001
*Syzygium aromaticum*	2.11	1.04–3.38	1.85 ± 0.67	19.35	<0.0001
Cypermethrin	0.05 × 10^−3^	0.02 × 10^−3^–0.54 × 10^−3^	2.09 ± 0.94	20.68	<0.0001
**Female**					
*Leptospermum citratum*	5.22	3.18–7.66	1.56 ± 0.43	23.53	<0.0001
*Leptospermum ericoides*	1.23	0.75–2.17	1.72 ± 0.54	38.70	<0.0001
*Leptospermum scoparium*	1.10	0.60–1.86	1.37 ± 0.38	33.40	<0.0001
*Kunzea ambigua*	16.94	9.07–na	1.32 ± 0.83	2.70	0.100
*Pimenta dioica*	3.55	1.88–5.41	1.39 ± 0.38	20.36	<0.0001
*Syzygium aromaticum*	3.53	2.07–5.20	1.80 ± 0.50	25.73	<0.0001
Cypermethrin	0.06 × 10^−3^	0.02 × 10^−3^–0.12 × 10^−3^	1.51 ± 0.52	18.64	<0.0001

CL: confident limit, na: not available.

**Table 4:** LD_50_ values for the non-polar and polar chromatographic fractions of *L. ericoides* and *L. scoparium* and the triketone fraction of *L. scoparium* against SWD.

Essential Oil	LD_50_ (µg/fly)	95% CL (µg/fly)	Slope ± SE	Effect Test	
				χ^2^	*P*
**Male**					
*Leptospermum ericoides* (NF)	24.83	0.07–na	0.33 ± 0.60	0.31	0.58
*Leptospermum ericoides* (PF)	0.37	0.19–0.69	1.07 ± 0.31	27.77	<0.0001
*Leptospermum scoparium* (NF)	7.25	3.07–17.14	0.89 ± 0.63	2.13	0.14
*Leptospermum scoparium* (PF)	0.38	0.21–0.67	1.37 ± 0.41	32.97	<0.0001
Triketone fraction (97.1%)	0.13	0.05–0.24	1.91 ± 0.80	23.42	<0.0001
**Female**					
*Leptospermum ericoides* (NF)	86.99	8.61–na	0.48 ± 0.84	0.34	0.56
*Leptospermum ericoides* (PF)	0.65	0.38–1.15	1.52 ± 0.45	38.55	<0.0001
*Leptospermum scoparium* (NF)	22.19	6.23–na	0.59 ± 0.69	0.78	0.38
*Leptospermum scoparium* (PF)	0.57	0.34–0.98	1.75 ± 0.56	40.28	<0.0001
Triketone fraction (97.1%)	0.22	0.13–0.39	2.16 ± 0.82	34.14	<0.0001

CL: confident limit, na: not available, NF: non-polar fraction, PF: polar fraction.

**Table 5:** List of tested essential oils.

Essential Oil	Scientific Name	Extraction Part	Origin	Source
*Leptospermum citratum* organic	*Leptospermum citratum (=L. petersonii)*	Blossoms	Australia/Tasmania	Oshadhi
Kanuka	*Leptospermum ericoides (=Kunzea ericoides)*	Leaves	South Africa	Oshadhi
Manuka	*Leptospermum scoparium*	Leaves	New Zealand	Oshadhi
Kunzea	*Kunzea ambigua*	Leaves	Australia	La Drome
Allspice	*Pimenta dioica*	Berries	Jamaica	Oshadhi
Clove bud	*Syzygium aromaticum*	Bud	Madagascar	La Drome

## References

[1] Asplen M.K., Anfora G., Biondi A., Choi D.S., Chu D., Daane K.M., Gibert P., Gutierrez A.P., Hoelmer K.A., Hutchison W.D (2015). Invasion biology of spotted wing Drosophila (*Drosophila suzukii*): A global perspective and future priorities. J. Pest Sci..

[2] Cini A., Anfora G., Escudero-Colomar L.A., Grassi A., Santosuosso U., Seljak G., Papini A. (2014). Tracking the invasion of the alien fruit pest *Drosophila suzukii* in Europe. J. Pest Sci..

[3] Lasa R., Tadeo E. (2015). Invasive drosophilid pests *Drosophila suzukii* and *Zaprionus. indianus* (Diptera: Drosophilidae) in Veracruz, Mexico. Fla. Entomol..

[4] Hauser M. (2011). A historic account of the invasion of *Drosophila suzukii* (Matsumura) (Diptera: Drosophilidae) in the continental United States, with remarks on their identification. Pest Manag. Sci..

[5] Goodhue R.E., Bolda M., Farnsworth D., Willams J.C., Zalom F.G. (2011). Spotted wing drosophila infestation of California strawberries and raspberries: Economic analysis of potential revenue losses and control. Pest Manag. Sci..

[6] Lee J.C., Dreves A.J., Cave A.M., Kawai S., Isaacs R., Miller J.C., Timmeren S.V., Bruck D.J. (2015). Infestation of wild and ornamental noncrop fruits by *Drosophila suzukii* (Diptera: Drosophilidae). Ann. Entomol. Soc. Am..

[7] Bruck D.J., Bolda M., Tanigoshi L., Klick J., Kleiber J., DeFrancesco J., Gerdeman B., Spitler H. (2011). Laboratory and field comparisons of insecticides to reduce infestation of *Drosophila suzukii* in berry crops. Pest Manag. Sci..

[8] Van Timmeren S., Isaacs R. (2013). Control of spotted wing drosophila, *Drosophila suzukii*, by specific insecticides and by conventional and organic crop protection programs. Crop. Prot..

[9] Knoll V., Ellenbroek T., Romeis J., Collatz J. (2017). Seasonal and regional presence of hymenopteran parasitoids of *Drosophila* in Switzerland their ability to parasitize the invasive *Drosophila suzukii*. Sci. Rep..

[10] Jang M., Kim J., Yoon K.A., Lee S.H., Park C.G. (2017). Biological activities of Myrtaceae plant essential oils and their major components against *Drosophila suzukii* (Diptera: Drosophilidae). Pest Manag. Sci..

[11] Park C.G., Jang M., Yoon K.A., Kim J. (2016). Insecticidal and acetylcholinesterase inhibitory activities of Lamiaceae plant essential oils and their major components against *Drosophila suzukii* (Diptera: Drosophilidae). Ind. Crops Prod..

[12] Kim J., Jang M., Shin E., Kim J., Lee S.H., Park C.G. (2016). Fumigant and contact toxicity activity of 22 wooden essential oils and their major components against *Drosophila suzukii* (Diptera: Drosophilidae). Pestic. Biochem. Physiol..

[13] Kim J., Jang M., Lee K.T., Yoon K.A., Park C.G. (2016). Insecticidal and enzyme inhibitory activities of sparassol and its analogs against *Drosophila suzukii*. J. Agric. Food Chem..

[14] Isman M.B. (2000). Plant essential oils for pest and disease management. Crop. Prot..

[15] Nerio L.S., Olivero-Verbel J., Stashenko E. (2010). Repellent activity of essential oils: A review. Bioresour. Technol..

[16] El-Seedi H.R., Khalil N.S., Azeem M., Taher E.A., Göransson U., Pålsson K., Borg-Karlson A.K. (2012). Chemical composition and repellency of essential oils from four medicinal plants against *Ixodes ricinus* (L.) nymphs (Acari: Ixodidae). J. Med. Entomol..

[17] Isman M.B., Miresmailli S., Machial C. (2011). Commercial opportunities for pesticides based on plant essential oils in agriculture, industry and consumer products. Phytochem. Rev..

[18] Priestley C.M., Williamson E.M., Wafford K.A., Sattelle D.B. (2003). Thymol, a constituent of thyme essential oil, is a positive allosteric modulator of human GABA_A_ receptors and a homo-oligomeric GABA receptor from *Drosophila melanogaster*. Br. J. Pharmacol..

[19] Enan E. (2001). Insecticidal activity of essential oils: Octopaminergic sites of action. Comp. Biochem. Physiol. Part. C: Toxicol. Pharmacol..

[20] Tong F., Gross A.D., Dolan M.C., Coats J.R. (2013). Phenolic monoterpenoid carvacrol inhibits the binding of nicotine to the housefly nicotinic acetylcholine receptor. Pest Manag. Sci..

[21] Tong F., Coats J.R. (2012). Quantitative structure–activity relationships of monoterpenoid binding activities to the housefly GABA receptor. Pest Manag. Sci..

[22] Renkema J.M., Wright D., Buitenhuis R., Hallett R.H. (2016). Plant essential oils and potassium metabisulfite as repellents for *Drosophila suzukii* (Diptera: Drosophilidae). Sci. Rep..

[23] Park H.M., Kim J., Chang K.S., Kim B.S., Yang Y.J., Kim G.H., Shin S.C., Park I.K. (2011). Larvicidal activity of Myrtaceae essential oils and their components against *Aedes aegypti*, acute toxicity on *Daphnia magna*, and aqueous residue. J. Med. Entomol..

[24] Lee Y.S., Kim J., Shin S.C., Lee S.G., Park I.K. (2008). Antifungal activity of Myrtaceae essential oils and their components against three phytopathogenic fungi. Flavour. Frag. J..

[25] Van Vuuren S.F., Docrat Y., Kamatou G.P.P., Viljoen A.M. (2014). Essential oil composition and antimicrobial interactions of understudied tea tree species. S. Afr. J. Bot..

[26] Van Den Dool H., Kratz P.D. (1963). A generalization of the retention index system including linear temperature programmed gas-liquid partition chromatography. J. Chromatogr. A.

[27] Porter N.G., Wilkins A.L. (1999). Chemical, physical and antimicrobial properties of essential oils of *Leptospermum scoparium* and *Kunzea ericoides*. Phytochemistry.

[28] Batish D.R., Singh H.P., Kohli R.K., Kaur S. (2008). Eucalyptus essential oil as a natural pesticide. For. Ecol. Manag..

[29] Park I.K., Kim J., Lee S.G., Shin S.C. (2007). Nematicidal activity of plant essential oils and components from Ajowan (*Trachyspermum. ammi*), Allspice (*Pimenta dioica*) and Litsea (*Litsea cubeba*) essential oils against pine wood nematode (*Bursaphelenchus xylophilus*). J. Nematol..

[30] Seo S.M., Kim J., Lee S.G., Shin C.H., Shin S.C., Park I.K. (2009). Fumigant antitermitic activity of plant essential oils and components from ajowan (*Trachyspermum ammi*), allspice (*Pimenta dioica*), caraway (*Carum carvi*), dill (*Anethum graveolens*), geranium (*Pelargonium graveolens*), and litsea (*Litsea cubeba*) oils against Japanese termite (*Reticulitermes speratus* Kolbe). J. Agric. Food Chem..

[31] Park I.K., Shin S.C. (2005). Fumigant activity of plant essential oils and components from garlic (*Allium sativum*) and clove bud (*Eugenia caryophyllata*) oils against the Japanese termite (*Reticulitermes speratus* Kolbe). J. Agric. Food Chem..

[32] Tian B.L., Liu Q.Z., Liu Z.L., Wang J.W. (2015). Insecticidal potential of clove essential oil and its constituents on *Cacopsylla chinensis* (Hemiptera: Psyllidae) in laboratory and field. J. Econ. Entomol..

[33] Waliwitiya R., Isman M.B., Verno R.S., Riseman A. (2005). Insecticidal activity of selected monoterpenoids and rosemary oil to *Agriotes obscurus* (Coleoptera: Elateridae). J. Econ. Entomol..

[34] Erland L.A.E., Rheault M.R., Mahmoud S.S. (2015). Insecticidal and oviposition deterrent effects of essential oils and their constituents against the invasive pest *Drosophila suzukii* (Matsumura) (Diptera: Drosophilidae). Crop. Prot..

[35] Christoph F., Kaulfers P.M., Stahl-Biskup E. (2000). A comparative study of the in vitro antimicrobial activity of tea tree oils *s.l.* with special reference to the activity of β-triketones. Planta Med..

[36] Reichling J., Koch C., Stahl-Biskup E., Sojka C., Schnitzler P. (2005). Virucidal activity of a β-triketone-rich essential oil of *Leptospermum scoparium* (Manuka oil) against HSV-1 and HSV-2 in cell culture. Planta Med..

[37] Jeong E.Y., Kim M.G., Lee H.S. (2009). Acaricidal activity of triketone analogues derived from *Leptospermum scoparium* oil against house-dust and stored-food mites. Pest Manag. Sci..

[38] Fang F., Candy K., Melloul E., Bernigaud C., Chai L., Darmon C., Durand R., Botterel F., Chosidow O., Izri A. (2016). In vitro activity of ten essential oils against *Sarcoptes scabiei*. Parasit. Vectors.

[39] Duke S.O., Dayan F.E., Romagni J.G., Rimando A.M. (2000). Natural products as sources of herbicides: current status and future trends. Weed Res..

[40] Dayana F.E., Duke S.O., Sauldubois A., Singh N., McCurdy C., Cantrella C. (2007). *p*-Hydroxyphenylpyruvate dioxygenase is a herbicidal target site for β-triketones from *Leptospermum scoparium*. Phytochemistry.

[41] Dumas E., Giraudo M., Goujon E., Halma M., Knhili E., Stauffert M., Batisson I., Besse-Hoggan P., Bohatier J., Bouchard P. (2017). Fate and ecotoxicological impact of new generation herbicides from the triketone family: An overview to assess the environmental risks. J. Hazard. Mater..

[42] Dalton D.T., Walton V.M., Shearer P.W., Walsh D.B., Caprile J., Isaacs R. (2011). Laboratory survival of *Drosophila suzukii* under simulated winter conditions of the Pacific Northwest and seasonal field trapping in five primary regions of small and stone fruit production in the United States. Pest Manag. Sci..

[43] Van Klink J.W., Brophy J.J., Perry N.B., Weavers R.T. (1999). β-Triketones from Myrtaceae: Isoleptospermone from *Leptospermum scoparium* and Papuanone from *Corymbia dallachiana*. J. Nat. Prod..

[44] Perry N.B., Van Klink J.W., Brennan N.J., Harris W., Anderson R.E., Douglas M.H., Smallfield B.M. (1997). Essential oils from New Zealand manuka and kanuka: chemotaxonomy of *Kunzea*. Phytochemistry.

[45] Brophy J.J., Goldsack R.J., Forster P.I., Clarkson J.R., Fookes C.J.R. (1996). Mass spectra of some β-triketones from australian Myrtaceae. J. Essent. Oil Res..

[46] Abbott W.S. (1925). A method of computing the effectiveness of an insecticide. J. Econ. Entomol..

